# Role of Parathyroid Hormone Assay and Bedside Ultrasound in the Emergency Department in Differentiating Acute Kidney Injury from Chronic Kidney Disease: A Systematic Review

**DOI:** 10.1155/2019/2102390

**Published:** 2019-03-12

**Authors:** Deepali Junnarkar Roy, Shrikant Digambarrao Pande, Zhong Hong Liew, Debajyoti Roy

**Affiliations:** ^1^Department of Emergency Medicine, Changi General Hospital, 2 Simei St 3, Singapore, Singapore; ^2^Department of Rehabilitation Medicine, Changi General Hospital, Singapore, Singapore; ^3^Department of Renal Medicine, Changi General Hospital, Singapore, Singapore

## Abstract

**Introduction:**

It is not uncommon for patients without preceding history of kidney disease to present to the Emergency department with renal failure. The absence of prior medical records or renal imaging presents a diagnostic challenge. Elevated parathyroid hormone levels or echogenic contracted kidneys on ultrasound are known to point to a diagnosis of chronic kidney disease. The literature in this regard is surprisingly limited. The objective of this study is to assess the role of intact parathyroid (iPTH) blood level and bedside ultrasound in differentiating acute kidney injury from chronic kidney disease.

**Methods:**

A systematic review which included a literature search of 3 databases, PubMed, Embase, and Cinahl (R) as also secondary sources, was done. The inclusion criteria evaluated studies which evaluated iPTH or bedside ultrasound in differentiating acute kidney injury from chronic kidney disease. We excluded studies which used other laboratory biomarkers like neutrophil gelatin associated lipocalin (NGAL) or carbamylated haemoglobin. A total of 2256 articles were identified. After screening, the relevant articles were reviewed, and an assessment of their methodological quality was made based on the CASP: Critical Appraisals Skill Programme.

**Results:**

Of the 2256 articles identified, after screening, only 5 were identified as relevant.

**Conclusions:**

An elevated parathyroid hormone level and echogenic contracted kidneys on bedside ultrasound in the Emergency department can help differentiate acute kidney injury from chronic kidney disease. This differentiation helps decide need for admission as well as further management. Although iPTH level may also rise in acute kidney injury, the value (2.5 times normal) can discriminate it from chronic kidney disease.

## 1. Introduction

In the Emergency department, it is increasingly common to find patients presenting for the first time with raised creatinine and no antecedent blood reports which could help differentiate acute kidney injury (AKI) from chronic kidney disease (CKD). This is specially a problem for patients who are temporary immigrants or tourists where previous medical records are unavailable. The differentiation of AKI from CKD is of paramount importance as it influences management, clinical course, prognosis, and disposition of the patient. AKI is a medical emergency and appropriate initial treatment can prevent complications and subsequent need for long-term renal replacement therapy [1, 2].

Often in these situations, it is unclear whether a patient has AKI or CKD or an AKI on the background of CKD. This can be a diagnostic dilemma for Emergency care team and also causes great apprehension for patients and their families. Current estimates suggest 8-16% of the world population has CKD. The supply of nephrologists is outstripped by this burgeoning demand. Emergency care physicians and internists will have to be conversant in this area.

The objective of our study is to assess the role of intact parathyroid (iPTH) blood level and bedside ultrasound in the Emergency room to differentiate acute kidney injury from chronic kidney disease based on a systemic review of literature.

## 2. Methods

To identify relevant studies, a literature search was conducted from 1966 up to 1^st^ January 2018. The search terms used and applied are described in the Appendix.

Ethics committee approval was not required as study did not involve human subjects or medical records. Literature search was limited to human studies in English language literature. Reference list of identified articles were manually searched for additional literature.

*The search strategy was applied to the following*:

1. MEDLINE (PUBMED): 1966 – 1^st^ January 2018
2. EMBASE; 1966- 1st January 2018
3. CINAHL (R): 1984 - 1st January 2018.

*Additional sources included in the secondary search strategy were*

1. references from relevant articles
2. Web-based resources:
  - http:///www.bestbets.org
  - http://www.clinicaltrials.gov
  - https://scholar.google.com.sg

## 3. Inclusion Criteria

All articles using intact parathyroid hormone level iPTH or ultrasound to differentiate AKI from CKD were included.

## 4. Exclusion Criteria

Trails using other laboratory parameters like (NGAL) or carbamylated haemoglobin to differentiate AKI and CKD were excluded. Records identified through EMBASE, MEDLINE, and CINAHL were identified for duplicates and excluded. Detailed search strategies for these 3 major database search engines are given in the Appendix. A uniform filter strategy was applied to all the major database engines.

## 5. Study Selection

The studies were initially screened to check against all titles and abstracts with regard to the eligibility criteria at Level I screening. Those publications not excluded in Level 1 screening were reviewed by 2 reviewers to confirm all eligibility criteria were met and no exclusions were applicable (Level 2).

## 6. Results of Literature Search

**Figure 1:**
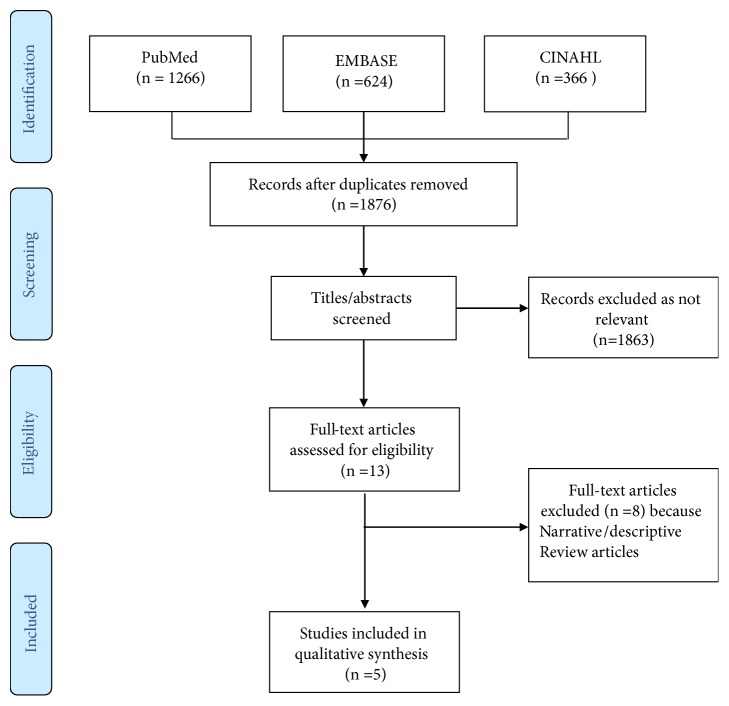
Flowchart of included and excluded trials in this clinical topic review.

There is a paucity of articles on this subject and hence all the articles which compared ultrasound and/or intact parathyroid hormone assay to differentiate AKI from CKD were included. Total 2256 articles were found but only 5 were relevant (Figure 1).

## 7. Assessment of Methodological Quality

The relevant articles were reviewed, and an assessment of their methodological quality was made based on the CASP: Critical Appraisals Skill Programme diagnostic tool questionnaire was used [3]. Assessment of study quality is summarised in Table 1. The general assessment of the review is in accordance with Guidelines for Systematic Reviews as previously described [4].

## 8. Discussion

The review of literature supports use of kidney ultrasound and/or measurement of serum iPTH levels in differentiating AKI from CKD in patients with deranged kidney function with no available prior serum creatinine values. However, there is paucity of literature in this area. The identified relevant studies were appraised and presented as per the guidelines for systematic reviews.

Factors which may help to differentiate AKI from CKD were identified. A history of nocturia and pruritus along with clinical features like anaemia, sallow skin, hypertension, or peripheral neuropathy would suggest CKD. A history of oliguria or anuria after a renal insult and normal sized kidneys on ultrasound with prior renal function suggesting a normal serum creatinine would suggest AKI. Unfortunately, in a large number of patients, the history is not available and other features have poor predictive value [2].

For the purposes of this study, AKI was defined as an increase of serum creatinine by at least one and a half times baseline as per the RIFLE criteria [5]. CKD was defined as structural or functional damage to the kidney or glomerular filtration rate (GFR) <60ml/minute for 3 months or greater [6]. In the absence of prior medical records and biochemical tests, ultrasound of kidneys remains the gold standard in differentiating AKI from CKD [6].

Ozmen et al. [7] prospectively analysed a cohort of 127 patients with serum creatinine higher than 3mg/dl (265 umol/L) and assessed role of ultrasound examination of kidney to differentiate acute from chronic kidney disease.

The authors concluded that renal length in patients with CKD was significantly shorter 90±15 mm, than those with AKI, 112±14 mm (p<0.001). When compared to healthy adult volunteers (N=33), renal length was (107±6 mm) almost similar to those in AKI group.

The ROC analysis curve for renal length cut off to differentiate AKI from CKD was 0.865.

A small kidney with highly echogenic parenchyma on ultrasound is characteristic of chronic renal failure/or chronic kidney disease [7]. Normal sized kidneys with normal or mildly increased echogenicity may indicate less severe disease. Renal parenchymal echogenicity is graded on a four-level scale using the normal liver or spleen as reference [8].

In patients with AKI, grade 1 echogenicity of the cortex was present in 33/62 (53%) patients. Of 65 patients with CKD, Grade 2 and grade 3 patients' echogenicity was seen in 34 (52%). Therefore, although the increase in echogenicity has less value as compared to renal length in differentiating AKI from CKD, hyperechogenic (Grade 3) kidneys were only seen in CKD [7].

Bennidor and Israelit [9] in a review of 137 adult patients with AKI with a rise of creatinine > 50% [0.3 mg/dl (26umol/L)] from baseline reported the use of renal ultrasound in Emergency department for evaluation of acute kidney injury. They excluded patients with no baseline kidney functions. 121 of the 137 patients with AKI (88.3%) had a normal renal ultrasound, suggesting normal length and echogenicity. 16 of the 137 patients (11.7%) had obstructive aetiology for the AKI. The limitations of this study were the small size, single centre, and retrospective nature.

Point of care ultrasound (POCUS) is widely used in the Emergency department. In fact, comprehensive training in POCUS is an integral part of Emergency medicine (EM) training in North America. When compared to standard consultative ultrasound, POCUS performed by the EM physician who knows the clinical history and examination can rapidly integrate ultrasound findings to better arrive at a management plan. EM physicians are already routinely performing renal ultrasound on patients with AKI, urinary tract infection, and nephrolithiasis [10]. A recent study comparing POCUS with conventional ultrasound and CT scan in suspected nephrolithiasis found no difference with respect to diagnostic accuracy, readmission rates, or complications [11].

Laboratory tests like carbamylated haemoglobin and serum 1,5-anhydroglucitol have been utilized to differentiate AKI from CKD [12, 13]. These tests are not used in clinical practice because of cost and availability. CKD results in derangement of the calcium, phosphate, and vitamin D homeostasis. This leads to low vitamin D levels, elevated serum phosphate, and consequent increase in parathyroid hormone synthesis (PTH) [14–16]. Patients with AKI may have an increase in intact PTH (iPTH) over a few days as a result of hypocalcaemia, hyperphosphatemia, and disordered vitamin D metabolism; the magnitude of rise may help differentiate AKI from CKD.

Ozmen et al. [2] prospectively studied iPTH levels in 122 patients with renal failure, those with AKI (n =64) and CKD (n=58). The diagnosis of AKI or CKD was based on relevant medical history, previous serum creatinine measurements, renal size on ultrasound, and radiological and clinical evidence of renal osteodystrophy. The ROC curve analysis was performed to investigate the role of iPTH in differentiating AKI from CKD. Area under the curve for iPTH was 0.92. They further found with an iPTH cut-off set at 170 pg/ml the sensitivity, specificity, positive predictive value, and negative predictive value to discriminate CKD were 88%, 89%, 88%, and 89%, respectively. Calculation of positive and negative likelihood ratio of 8 and 0.1 would have made the study more robust as it translates the characteristic of iPTH into clinical significance.

Cavayero et al. [17] described a small case series, 6 patients with elevated serum creatinine, AKI-4 and CKD-2 who had serum iPTH levels assayed. They concluded that iPTH was an inexpensive and readily available marker for differentiating AKI from CKD. The authors accept the small sample size; infrequent iPTH assessments were limitations. Findings similar to this study were reported by Parmar et al. [18].

Zhang et al. in their systemic review and meta-analysis concluded that serum Cystatin C (Cys C) appears to be a good biomarker in the prediction of AKI while urinary Cys C excretion had only moderate diagnostic value [19].

## 9. Conclusions

Physicians working in acute care, Emergency medicine, or primary care frequently encounter patients with undifferentiated renal failure and no prior medical records. Early differentiation between AKI and CKD can benefit the management of this group of patients. Available literature supports the use of kidney ultrasound and serum iPTH assays. The strength of evidence is moderate. Further studies are required based in the Emergency department which would validate whether point of care ultrasound is useful in the above scenario.

It is important to remember that iPTH level may rise in acute kidney injury; however, a cut-off value set at 170ng/ml is an excellent discriminator from chronic kidney disease.

## Figures and Tables

**Table 1:** Study Quality Assessment.

Author, year, country, study	Level of evidence/Study design Participants/Inclusion criteria	Intervention and Control Groups	Key outcome and results	Critical appraisal, Results
Ozmen CA et al, 2010 Turkey (Title: *Ultrasound as a diagnostic tool to differentiate acute from chronic renal failure.)* Clinical Nephrology, Vol. 74 – No. 1/2010 (46-52)	Level of evidence: II Prospective Observational cohort study. Study compares Sonographic results of patients with ARF and CKD and controls. 127 consecutive patients with serum creatinine >3 milligrams(mg) per decilitre(dl) and 33 healthy volunteers (i) Renal length along longest axis of the left and right kidney measured and mean calculated. Echo intensity of the cortex measured as Grades I to III which is comparison with echogenicity of liver	127 patients with serum creatinine level >3mg/dl 33 Health workers with no known Renal disease	*Group AKI* N=62 (i) Renal length 112+/-14mm (ii) Cortical echogenicity Grade 0: 19 Grade I: 33 Grade II: 10 Grade III: 0 *Group CKD* N=65 (i) Renal length 90±15mm (ii) Cortical echogenicity Grade 0: 4 Grade I: 32 Grade II: 27 Grade III: 7 *Group C Control* n= 33 (i) Renal length 107+/-6mm (ii) Cortical echogenicity Grade 0: 28 Grade I: 5 Grade II: 0 Grade III: 0	(i) P value calculated. (ii) ROC (Receiver operating characteristic curve ) analysis performed (iii) Area under curve AUC for parenchymal thickness is 0.724 (iv) AUC for renal length is 0.873 (v) Statistical methods explained. Student T test for parametric values, Chi 2 test for frequencies were used. (vi) Small study, single centre, (vii) Radiologists performing ultrasound were blinded.

Chase Canavero et al, 2015, USA (Title: *Blast from the past- using PTH to differentiate acute versus chronic kidney disease*) J Nephrol Ther 2015, 5:1	Level of evidence: IV Case series Reports on 6 patients with either acute kidney injury or Chronic kidney disease Intact parathyroid hormone assay measured in renal failure. Cases followed and blood tests: Creatinine, calcium, phosphorus, potassium, iPTH repeated.	Case series of 6 patients with either acute kidney injury or Chronic kidney disease. (i) AKI: 4 patients (ii) CKD: 2 Patients	*Group AKI: n= 4* do not have persistent high iPTH level *Group CKD: n= 2* High creatinine observed in CKD is associated with a persistent elevated PTH level.	(i) Case series (ii) Small sample size. (iii) iPTH level showed Sensitivity of 88% & Specificity of 89% (iv) Single centre, no uniform protocol, no p value calculated (v) Weakness: iPTH level repeated only in 2 cases and trend of the parathyroid hormone not well established. (vi) Limitation and further scope clearly mentioned.

S Ozmen R Danis et al, 2007, Turkey (Title: *Parathyroid hormone as a marker for the differential diagnosis of acute and chronic renal failure*.) Renal Failure, 29:509–512, 2007	Level of evidence: II Study design: Prospective observational study To establish the potential role of iPTH as a marker for a differential diagnosis of AKI and CKD compared with diagnosis based on relevant past medical history, radiological findings and lab tests Inclusion criteria: Prospective cohort 122 patients with serum creatinine > 2 milligram(mg) per decilitre (dl)	(i) AKI n=64, (ii) CKD n=58	A cut off for intact parathyroid hormone at 170 picogram (pg) per millilitre (ml) (i) High sensitivity (88%) (ii) High specificity (89%) (iii) Positive predictive value 88% (iv) negative predictive value of 89% (v) AUC for iPTH 0.92 *Group AKI n*=64 iPTH (pg/mL): 102 ± 64 Serum creatinine (mg/dL): 6.3 ± 4.2 Serum BUN (mg/dL): 89 ± 34 *Group CKD* n= 58 iPTH (pg/mL): 430 ± 280 Serum creatinine (mg/dL): 7.7 ± 4.1 Serum BUN (mg/dL): 91 ± 40	(i) Prospective trial with clear protocol. (ii) Sample size calculated at a significance level of 5%, power of 80% and assumption of sensitivity of 80-95%. (iii) Statistical methods explained. Student T test for parametric values. Chi 2 test for frequencies used. (iv) Sampling technique NOT mentioned. (v) Inclusion and exclusion criteria NOT mentioned. (vi) Results summarized as Sensitivity, specificity, PPV. +ve (8) or –ve (0.1) LR Not calculated. (vii) In ROC analysis AUC = 0.92 Single centre study mentions the need for Large multicentre RCT.

Bennidor Raviv, 2014, Israel (Title: *Renal ultrasound in the evaluation of acute kidney injury in the emergency department)* American Journal of Clinical Medicine Research, 2014, Vol. 2, No. 5, 103-105	Level of evidence= III Retrospective study Aim of the study is (i) To evaluate effectiveness of ultrasound in the evaluation of AKI in Emergency Department (ii) To find out stratifying factors that will help to identify patients who will be benefitted by ultrasound.	(i) n = 137 Male: female:77:56 mean age 70 years (ii) Inclusion criteria: 18 years old and with AKI identified as rise of at least 50% in baseline creatinine or at least 0.3 mg% from baseline creatinine	(i) Ultrasound defined as pathologic: if demonstrated obstructive aetiology for AKI. (ii) Normal US 121; Pathologic US: 16 (iii) Normal US group: Serum creatinine 3.88 + 2.84 Pathologic US group serum creatinine 4.30 +4.49 (iv) 11.7 % of AKI patients identified as due to obstructive renal failure	Material and methods explained in detail. Table 2 describes the influence of independent parameters on ultrasound result. There is no foot note to explain symbols. Results need more and clear explanation. Logistic regression method mentioned in materials and methods but not reported. Results in text and tables match Limitation: small study, wide confidence interval Suggested large multicentre prospective study

M Winston et al, 1977, Los Angeles, California USA (Title: *Ultrasonography in the management of unexplained renal failure)* Journal of Clinical Ultrasound,1978, Vol 6, 1-72	Level of evidence: IV Case series Ultrasound imaging as a first diagnostic procedure in acute unexplained renal failure to identify obstructive component and the potential for recovery.	Case series of 7 patients	Patients identified as CKD, Polycystic kidney, Bilateral hydro nephrosis, bladder tumour, cancer prostate	(i) Small number of cases analysed. (ii) No impact of cost analysed (iii) No uniform protocol in the study (iv) Narrative description of cases (v) No sensitivity or specificity calculated. (vi) Single centre, no uniform protocol, no p value calculated

## Data Availability

Data are available upon request.

## Appendix

### A. Database Search Strategy

*Embase*. 'acute kidney failure'/exp OR 'acute kidney failure' OR 'acute kidney injury'/exp OR 'acute kidney injury' OR aki OR 'acute renal failure'/exp OR 'acute renal failure' OR 'chronic kidney failure'/exp OR 'chronic kidney failure' OR 'chronic renal failure'/exp OR 'chronic renal failure' OR 'end stage renal failure'/exp OR 'end stage renal failure' OR 'end stage renal disease'/exp OR 'end stage renal disease' OR esrf OR 'esrd'/exp OR esrd OR 'uremia' OR 'uremia'/exp OR uremia OR 'azotemia' OR 'azotemia'/exp OR azotemia AND (diagnos*∗* OR differentia*∗*) AND ('ultrasound' OR 'ultrasound'/exp OR ultrasound OR 'ultrasonography' OR 'ultrasonography'/exp OR ultrasonography OR usg OR us OR 'parathyroid hormone'/exp OR 'parathyroid hormone' OR 'intact parathyroid hormone' OR ipth OR 'pth'/exp OR pth) AND 'diagnosis'/lnk AND ('human'/exp OR 'human') AND ('cohort analysis'/exp OR 'cohort analysis' OR 'comparative study'/exp OR 'comparative study' OR 'cross-sectional study'/exp OR 'cross-sectional study' OR 'diagnostic test accuracy study'/exp OR 'diagnostic test accuracy study' OR 'multicenter study'/exp OR 'multicenter study' OR 'observational study'/exp OR 'observational study' OR 'prospective study'/exp OR 'prospective study' OR 'retrospective study'/exp OR 'retrospective study')

*PubMed*. (AKI OR “Chronic kidney failure”) OR “Chronic kidney disease”) OR “End stage renal failure”) OR “End stage renal disease”) OR ESRF) OR ESRD) OR Azotemia)) OR (“Kidney Failure, Chronic”*∗*Mesh+) OR “Renal Insufficiency”[Mesh]) OR “Acute Kidney Injury”[Mesh]) OR “Renal Insufficiency, Chronic”[Mesh] OR “Acute kidney injury”) OR “Acute kidney failure”) AND (Differentia*∗* OR Diagnos*∗*)OR (“Diagnosis”[Mesh]) OR “diagnosis” [Subheading]) AND (Ultrasound) OR Ultrasonography) OR USG) OR US) OR (“Ultrasonography”[Mesh]) OR “ultrasonography” [Subheading]) OR “Parathyroid Hormone”*∗*Mesh+) OR (“parathyroid hormone”) OR “intact parathyroid hormone”) OR iPTH) OR PTH) NOT (surgery OR cardi*∗*) Filters: Case Reports; Comparative Study; Multicenter Study; Observational Study; Humans; English

*CINAHL*. “Acute kidney failure” OR “Acute kidney injury” OR AKI OR ARF OR “Acute Renal Failure” OR “Chronic kidney failure” OR “Chronic kidney disease” OR “Chronic Renal Failure” OR CRF OR CKD OR “End stage renal failure” OR “End stage renal disease” OR ESRF OR ESRD OR Uremia OR Azotemia OR Azotaemia

“Kidney Failure, Chronic”[Mesh] OR “Renal Insufficiency”[Mesh] OR “Acute Kidney Injury”[Mesh] OR “Renal Insufficiency, Chronic”[Mesh]

Diagnos*∗* OR Differentia*∗* OR “Diagnosis”[Mesh] OR “diagnosis” [Subheading]

Ultrasound OR Ultrasonography OR USG OR US OR “Ultrasonography”[Mesh] OR ultrasonography” [Subheading]

“Parathyroid hormone” OR “intact parathyroid hormone” OR iPTH or PTH OR “Parathyroid Hormone”[Mesh]
